# Improving Guideline Adherence for the Radiological Investigation of Acute Renal Colic: A Two-Cycle Quality Improvement Project

**DOI:** 10.7759/cureus.73472

**Published:** 2024-11-11

**Authors:** Muhammad H Ali, Berk Abay, Ahmed Ali, Mohammed Saad, Munir Al-Ghazawi, Hussein Kamel

**Affiliations:** 1 Urology, Barts Health NHS Trust, London, GBR; 2 Emergency Medicine, Barts Health NHS Trust, London, GBR; 3 Radiology, Barts Health NHS Trust, London, GBR

**Keywords:** acute renal colic, clincal audit, ct kub, ionising radiation, urinary stones

## Abstract

Introduction

Renal colic is a frequent complaint presented to healthcare services. Delayed diagnosis can result in life-threatening complications, including renal failure and sepsis. Low-dose, non-contrast computerised tomography of the kidneys, ureters, and bladder (CT KUB) is regarded as the investigation of choice when investigating acute renal colic in the adult population, with a high sensitivity and specificity for detecting calculi.

This audit is aimed at improving guideline adherence in the radiological investigation of renal colic, regarding the prompt and regulated use of CT KUB to ensure an acceptable diagnostic yield of urinary calculi and minimal patient complications secondary to delayed diagnosis and radiation exposure.

Methods

A retrospective study was conducted for all patients referred to radiology for suspected renal colic from accident and emergency (A&E) and primary care. A first-cycle audit in June involved reviewing 78 patients’ records, of whom 75 (96%) were included in the study to assess for the use of appropriate imaging modality (CT KUB), the diagnostic yield of urinary calculi with CT KUB, and whether patients were investigated within 24 hours as per stipulated guidance. Data collected were analysed and presented to each department with recommendations, following which a second-cycle audit was undertaken in October to assess their impact. Eighty-one patients’ records were screened, with 75 (93%) included in our study.

Exclusions included pregnant women, those who are known, recurrent stone formers and had a CT KUB in the last three months, and those aged less than 18.

Results

The first cycle study revealed that only 42 (56%) patients were appropriately investigated for renal colic with CT KUB. Many patients faced significant delays, with only 50 (66.7%) investigated within 24 hours. Of those investigated with CT KUB, 17 (40.5%) scans were positive for urinary calculi. This demonstrated a significant deviation from the recommended standards in the guidelines.

Following a period of re-education, raising awareness of guidelines, and emphasising the importance of a thorough clinical history in scan requests, the second cycle demonstrated a significant improvement in the use of CT KUB to investigate renal colic, with the total reaching 55 (73.3%) patients, along with a concurrent increase in the proportion of patients with positive findings for calculi to 36 (65.5%). No significant improvement was demonstrated in the number of patients investigated within 24 hours, with 24 (32.0%) patients facing delay.

Conclusion

This two-cycle audit highlights the importance of timely and appropriate use of CT KUB in investigating renal colic. The first audit revealed significant delays and suboptimal adherence to institutional guidance. After educational intervention, the second cycle showed improved CT KUB utilisation towards the required standard, with an associated higher diagnostic rate of scans for urinary calculi (above the minimum of 44%-64%), reducing the likelihood of developing complications from missed diagnoses. Ongoing education in the form of posters and departmental lectures will ensure an increasing trajectory towards the 100% target for CT KUB use in investigating renal colic, and future efforts to reduce investigation delays will include introducing streamlined referral and communication pathways between primary and secondary care. A third-cycle study will assess the impact of these changes.

## Introduction

Renal colic refers to sudden onset, severe, and sharp flank pain secondary to the obstruction and distension of the urinary tract [1]. It may be described as radiating from 'loin to groin' and is often associated with nausea and vomiting. As a stone migrates, patients may describe difficulty passing urine and may also present with concurrent features of systemic upset, including tachycardia and pyrexia. The main cause of renal colic is ureterolithiasis [2].

Renal colic is an exceedingly common presentation to medical services, with a lifetime incidence of 12% in males, 6% in females, and a recurrence rate of 30%-50% if left untreated [3, 4]. Furthermore, there is an increasing prevalence of nephrolithiasis, with some studies reporting an incidence increase of 5% from the late 90s to the early 2000s [5].

While a common presentation, delayed and missed diagnoses can carry significant complications, including renal failure and sepsis [2]. Delayed decompression secondary to an obstructing stone can increase the odds of mortality by 29% [6]. Given the severe consequences of complications and the increasing prevalence of renal colic, it is essential patients are investigated promptly with an appropriate imaging modality that has been demonstrated to carry a good diagnostic yield.

The British Association of Urological Surgeons (BAUS) and the National Institute for Health and Care Excellence (NICE) recommend using low-dose, non-contrast computed tomography of the kidneys, ureters, and bladder (CT KUB) for investigating suspected renal colic within 24 hours of presentation. This recommendation is based on evidence indicating that CT KUB is more effective at detecting kidney stones than other modalities, including plain X-ray, ultrasonography, and magnetic resonance imaging (MRI) [7, 8]. Despite being a "low-dose" modality, CT KUB still involves notable radiation exposure, prompting the Royal College of Radiologists (RCR) to recommend auditing its use to ensure sufficient diagnostic value [9].

This two-cycle audit conducted across April and October 2024 was undertaken to assess adherence to guidelines at Newham University Hospital (NUH), a district general hospital in London, United Kingdom, concerning the radiological investigation of renal colic. Where deviations from guidance were noted in the first cycle, recommendations were implemented and reassessed in a second, closed-loop retrospective study to assess their impact on better diagnosis and patient outcomes.

## Materials and methods

This study represents a comprehensive retrospective analysis of CT KUB scans done for the investigation of renal colic at NUH. Permission was sought from the Clinical Effectiveness Unit at the hospital before accessing any patient records, ensuring ethical and methodological integrity. All data were anonymised to ensure identifiable information was erased to maintain confidentiality.

The study comprised two audit cycles, with the first audit surveying CT KUB scans done between April and June 2024 and the reaudit spanning from August to October 2024. For each phase, the data collection continued until the target sample size of 75 patients was reached.

Our study excluded pregnant women and patients under 18, as CT KUB is avoided in these groups due to the increased risks associated with radiation exposure. In children, heightened sensitivity to radiation and early-life exposure allows a greater window for potential long-term effects, such as malignancy, to develop [10]. Similarly, in pregnant women, foetal radiation exposure is associated with an increased risk of teratogenicity and childhood malignancy; as a result, guidelines recommend ultrasound as the preferred initial imaging modality for both groups, and they have not been included in our cohort [8, 11].

For patients with a history of stone formation presenting with renal colic, the RCR and the BAUS recommend ultrasound or X-ray KUB if a CT KUB has already been conducted within the past three months as first-line imaging [7, 9]. This is in a bid to minimise cumulative radiation exposure from repeated CT scans, which is associated with a higher risk of developing malignancy [12]. Consequently, these patients were also excluded from our study, as CT KUB is not the recommended first-line investigation in this population.

A total of 78 patients were reviewed retrospectively in the first audit. Patient data analysed included patient demographics; details mentioned in the investigation request; source and date of investigation request; radiologist impression; and the time of scan to determine if a patient was investigated within 24 hours. One pregnant patient, one younger than 18, and one recurrent stone former with CT KUB in the last three months were excluded from the first cycle, with our resultant sample of 75 patients consisting of 45 women (60.0%) and 30 men (40.0%).

The data were then compared against the professional standards set by NICE, BAUS, and the RCR. These state that 100% of patients, where exclusions do not apply, should be investigated with CT KUB; all patients presenting with renal colic should be investigated urgently within 24 hours; and a positive diagnosis of renal calculi must be made in 44%-64% of scans, with alternative diagnoses noted in a further 6%-18% [7-9].

To disseminate findings and raise awareness amongst relevant stakeholders, the results of the first cycle were presented at the hospital radiology governance meeting and to the emergency department. Recommendations were presented and implemented to improve the quality of care. These interventions were predominantly education-based, where refreshers were delivered for medical and allied staff, signposting to the relevant guidelines. Additionally, emphasis was also placed upon mentioning key clinical details suggestive of renal colic, elicited from thorough clinical assessment, to facilitate a robust vetting process. This information was also distributed amongst primary care providers using representatives.

Finally, we reaudited to determine the impact of our recommendations. A total of 81 patients were screened, and six patients were excluded from the second cycle in accordance with the above criteria. Our final sample consisted of 34 women (45.3%) and 41 men (54.7%). Data were collected and analysed in the same manner as above, under the supervision of a consultant radiologist to ensure integrity. Statistical analysis was conducted using Fisher's exact tests to determine the independence of categorical variables and independent t-tests to compare means across groups. The results were considered statistically significant if the p-value was <0.05.

## Results

Table 1 presents a detailed overview of the collected data, including patient demographics, the proportion of patients who underwent CT KUB for suspected renal colic, and the percentage of scans that confirmed urinary calculi in both cycles. Additionally, it highlights the number and proportion of delayed scans from each referring department, along with the average delay time. 

**Table 1:** Patient demographics and key results The table represents patients' demographics and key data with respect to our auditing standards. Data are expressed in numbers of patients (N), years (years)and percentage (%). P-value is significant at P<0.05. NS indicates an insignificant result; CT KUB: computed tomography of the kidney, ureter, and bladder

	First cycle	Second cycle	P-values
Total number of cases reviewed	78	81	-
Patients included (N)	75	75	-
Mean age (years)	42.34 (±12.94)	42.06 (±12.03)	0.89
Age range (years)	20 – 83	20 – 83	-
Females	45 (60.0%)	34 (45.3%)	-
Males	30 (40.0%)	41 (54.7%)	-
Underwent low-dose non-contrast CT KUB	42 (56.0%)	55 (73.3%)	0.04
Underwent ultrasonography	33 (44.0%)	20 (26.7%)	
CT KUB positive for urinary calculi	17 (40.5%)	36 (65.5%)	0.02
CT KUB alternative diagnosis	4 (9.52%)	6 (10.9%)	NS
Proportion of alternative imaging modality requests from primary care (%)	21 (63.6%)	12 (60.0% )	-
Delayed scans (not performed in 24 hours)	25 (33.3%)	24 (32.0%)	NS
Proportion of delays from primary care (%)	23 (92.0%)	21 (87.5%)	-
Average time of delay (days)	38.04	24.65	-

In the first audit, the mean age of patients was 42.34 years, with a standard deviation of 12.94. For the reaudit, the mean age was slightly lower at 42.06 years, with a standard deviation of 12.03. Both audits encompassed a wide age range spanning 20 to 83 years of age, demonstrating a diverse population. Sex distribution and mean age were similar across the two audit cohorts, indicating a consistent demographic profile between the two cycles.

In the first audit, a total of 78 patients were assessed, and 75 patients were included in the study. Notably, only 42 patients (56.0%) had a CT KUB scan for renal colic. This falls short of the standards set by the RCR, which state that 100% of patients suspected to have renal colic should have a CT KUB scan, where exclusions do not apply [9]. Of the remaining 33 patients (44.0%), 21 (63.6%) were referred from primary care.

Out of 42 CT KUB scans performed, 17 (40.5%) were positive for urinary tract calculi, while four (9.5%) scans demonstrated alternative pathology, all of which were non-urological. The findings on imaging were fibroids, common bile duct dilatation, mesenteric adenitis, and faecal loading. Although the latter percentage is within acceptable limits, the lower-than-expected proportion of positive scans, falling below the required standard of a minimum of 44%-64%, indicated an over-reliance on CT KUB to assess non-specific abdominal pain rather than as confirmation for suspected renal colic.

This was supported by our review of clinical histories included in requests for CT KUB to determine if imaging was indicated based on the information provided. Of the 42 CT KUB scans performed, we found 15 requests (35.7%), where details relayed were minimal. In some cases, this was limited to ‘right iliac fossa pain’, isolated symptoms of infection, unspecified abdominal pain, and a list of differential diagnoses with no accompanying history. In isolation, these symptoms would not usually warrant a CT KUB without further index of suspicion for renal colic, as they may indicate other pathology that may be best investigated with alternative methods. Consequently, only two (13.33%) scans revealed a stone, demonstrating a substantial disparity. Where we felt the information presented was more in keeping with renal colic, with the most common reason being loin-to-groin pain with associated haematuria, 15 (56%) out of the 27 identified requests (64.3%) had positive findings for calculi. This formed the basis of our education-focused approach, where we felt the importance of clinical history had to be stressed to both vetting radiologists and referring physicians.

Additionally, the first audit also showed that 25 patients (33.3%) were not investigated within 24 hours of presentation. The average delay was 38.04 days, which conveyed significant concern given the degree of deviation from the required standard. Twenty-three of these patients (92.0%) were referred for investigation from primary care.

**Figure 1:**
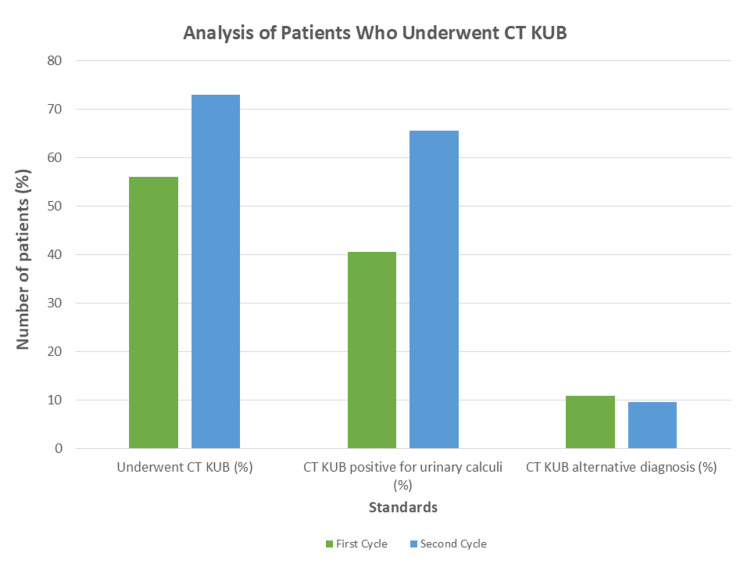
Analysis of Patients who Underwent CT KUB A comparison of the percentage of patients who underwent CT KUB, were diagnosed with a stone. and were diagnosed with alternative pathology; this figure demonstrates improvement towards the required standards for investigation with CT KUB. CT KUB: computed tomography of the kidney, ureter, and bladder

Following the implementation of changes and allowing time for the effect to be seen, the audit was repeated in October 2024. The reaudit included 75 patients after six were excluded due to exclusion criteria. We noted significant improvement in practice in two out of three standards. Figure 1 illustrates these changes.

In the second cycle, 55 patients (73.3%) underwent CT KUB for investigation of renal colic, a 17% increase that is statistically significant (p = 0.04). Furthermore, the proportion of CT KUB scans positive for calculi rose from 17 cases (40.5%) to 36 cases (65.5%), marking a 25% improvement that is also statistically significant (p = 0.02) and aligns with the RCR standard of a minimum detection rate of 44%-64%. This improvement can be attributed in part to a reduction in vague clinical histories accompanying CT KUB requests, allowing for a more thorough vetting process. Among the 55 scans performed, 42 (76.0%) included clear clinical information indicative of renal colic, with a concordantly higher positivity rate for calculi, with 33 scans (78.6%) confirming the presence of a stone compared to the first cycle. Conversely, when clinical history was vague, with details similar to the first cycle, out of 13 requests (24.0%), only one scan (7.69%) was positive for a stone. This highlights the importance of ongoing reinforcement of the need for detailed clinical histories to minimise harmful radiation exposure in cases where CT KUB may not be warranted.

Of the remaining 20 (26.7%) patients investigated with ultrasound, 12 (60.0%) were referred from primary care, demonstrating a consistent proportion between both cycles. Furthermore, there was no statistically significant improvement in the patients who were investigated within 24 hours. Twenty-four patients (32.0%) were not investigated on time for renal colic in the second cycle, compared to 25 (33.3%) in the first. Twenty-one (87.5%) of these patients were referred from primary care, demonstrating a large majority as in the first cycle. However, of note, there was an improvement in reducing the average time of delays, which fell from 38.04 to 24.65 days.

To summarise the positive outcomes, the percentage of patients receiving a CT KUB scan for renal colic increased from 42 (56.0%) to 55 (73.3%) (p = 0.04). The CT KUB scans that were positive for urinary tract calculi increased from 17 (40.5%) to 36 (65.5%), which is again a significant increase (p = 0.02). These findings underline the effectiveness of raising departmental awareness of guidelines through our education programme, and placing a greater emphasis on providing adequate clinical assessment details in investigation requests. This shift towards evidence-based practice, with increased use of CT KUB and improved detection rates for calculi, sets a premise for improvement in patient outcomes by facilitating earlier diagnosis and treatment.

## Discussion

In this quality improvement project, we aimed to improve clinical practice at NUH towards evidence-based practice as per NICE, BAUS, and RCR guidance. Specifically, we aimed to work towards three standards: 100% of patients suspected to have renal colic, where exclusions do not apply, should be investigated with CT KUB; all patients presenting with renal colic should be investigated within 24 hours; and a positive diagnosis of renal calculi must be made in 44-64% of scans, with alternative diagnoses noted in a further 6%-18% [9].

A CT KUB is the preferred investigation modality for renal colic according to current guidance, with a reported sensitivity of 98% and specificity of 97% [13]. One of the most frequently used alternative imaging modalities in the investigation of renal colic is ultrasound. A significant benefit of its use is no radiation exposure; indeed, this is why it is recommended as the primary mode of investigation in patient groups where exposure to radiation may result in significantly more adverse outcomes, including children and pregnant women [8]. However, its diagnostic accuracy in investigating renal colic is compromised by limitations such as operator proficiency and a propensity for overestimating stone size, which could alter management [14]. A pooled study assessing the use of imaging modalities in the investigation of renal colic reported a sensitivity and specificity of 84% and 53%, respectively, for detecting urinary calculi with ultrasonography. Plain film imaging also demonstrated lower accuracy than CT KUB, with sensitivity at 57% and specificity at 76%. Whilst MRI offers a higher specificity of 98%, its lower sensitivity of 82% and the associated higher costs make it a less practical option for routine use in renal colic diagnosis [15]. This offers some insight as to why CT KUB is advocated for in professional guidance.

A CT KUB offers further diagnostic advantages with management planning; it allows for parameters such as the density of urinary calculi to be calculated, which can guide treatment prognosis using methods such as extracorporeal shockwave lithotripsy (ESWL) [16]. Furthermore, image analysis for features such as ‘ureteral wall thickness’ and the absence of ‘rim sign’ on CT KUB, a 1-2mm soft-tissue attenuation surrounding a calculus, can be used alongside other parameters and clinical assessment to predict the likelihood of stone passage, helping inform conservative management decisions [17, 18]. These additional benefits, alongside well-documented diagnostic accuracy, provide context as to why the 100% target for investigation with CT KUB is so important.

It is imperative that patients presenting with signs and symptoms consistent with renal colic are investigated in line with current evidence-based practice, as a missed or delayed diagnosis can carry significant morbidity and mortality for patients. Complications include forniceal rupture with extravasation, pyelonephritis, renal failure, sepsis, and death [2, 6]. As a result, current advice is that patients are investigated within 24 hours to prevent the development of such complications [7-9].

While CT KUB offers valuable diagnostic advantages, it is not without associated risks. Research has demonstrated a dose-dependent relationship between radiation exposure and cancer risk, with repeated scans increasing the likelihood of developing both solid and haematological cancers [12]. Consequently, it is crucial to use CT KUB judiciously, ensuring that its diagnostic benefits outweigh the potential risks from radiation. The RCR recommends monitoring the diagnostic yield of CT KUB for kidney stones, with an expected detection rate of 44%-64%; if rates fall below this range, an audit of scan ordering practices is advised to ensure patients aren’t being unnecessarily exposed to radiation [9].

Where CT KUB is indicated, measures can be taken to minimise harm from radiation exposure by reducing the dose involved and restricting the field of exposure to where it is only absolutely necessary. These include using a low-dose CT KUB protocol, where the radiation dose is reduced without excessively compromising image quality, and limiting any excess scan length above the upper pole of the highest kidney to no more than 10% of the total scan length [9, 19]. These safeguards help ensure patient harm from radiation is minimised for all patients, but especially in those patients whose recurrent stone formation may warrant repeat investigation, provided the interval between re-presenting is sufficient enough to warrant CT imaging rather than initially with ultrasound or plain film [7].

Epidemiological data suggests that renal colic shows a higher incidence in males, with an average age between 40 and 50 years [20-22]. Patient demographics in the first cycle of our study differed slightly from published epidemiological data on renal colic, as our first-cycle cohort consisted mostly of females at 60% (45/75). However, our second cycle was more representative, with the majority consisting of males at 54.7% (41/75). The mean age for both cycles of our audit remained consistent, falling between 40 and 50 years of age (42.34 and 42.06, respectively).

In the first cycle of our study, we demonstrated deviation in practice with regard to the above standards, where only 42 patients (56.0%) were investigated with CT KUB; of these patients, 17 (40.5%) were diagnosed with a stone. From our analysis, we felt the main reasons for discrepancy were a lack of awareness of guidelines and an over-reliance on imaging to differentiate causes of non-specific abdominal pain rather than as a means to confirm suspected renal colic. Many imaging requests were vague and, in some cases, only consisted of a list of differential diagnoses, which lacked specificity for the most likely diagnosis with a complementing clinical history. This meant the vetting process was not as robust as intended, with many patients investigated with the incorrect imaging modality as a means to investigate the various suspected underlying causes.

We found other studies published in the literature that conveyed similar results. A study conducted by Himelfarb et al. reported only 27% of adult patients presenting with uncomplicated renal colic received CT imaging [23]. Two further studies conducted in the United Kingdom reported a positive CT KUB rate of 47.5% and 41.2%, respectively, with alternative diagnoses noted in 10% and 9.8% of patients [21, 24]. In line with our findings, these studies also mentioned the need for further improvement in investigation requests.

To address this, we organised an education programme. This spanned over two months with four repeated sessions, allowing participation for professionals with different rota responsibilities from not only the emergency and radiology departments but anybody with an interest. Before delivering our sessions, we distributed our findings to provide a background on our study and findings for context. The sessions consisted of a refresher, teaching physicians and allied health professionals about renal colic, including pathophysiology, clinical presentation, the recommended investigation modalities, and differential diagnoses. Emphasis was placed upon screening for symptoms we most frequently encountered on CT scans that were positive for urinary calculi that are also well documented in the literature, including sharp, colicky pain radiating from the loin to the groin, commonly associated with nausea, vomiting, and haematuria. We also addressed the presentation and possible differentiating factors in clinical assessment for common differentials that may present with similar features to renal colic, including pyelonephritis, diverticulitis, and gynaecological pathology. In all cases, we encouraged holistic assessment to help guide clinicians in cases where renal colic may present atypically, including assessing for risk factors such as hypercalcaemia and a family history of nephrolithiasis, where the presence of these features may increase the index of suspicion in these cases [2]. We encouraged the inclusion of the above features in investigation requests to ensure vetting radiologists have adequate information to determine if a patient is best investigated with CT KUB for urinary calculi or if other diagnoses are more likely that may warrant a different imaging technique.

Following this, we emphasised the standards set in the NICE, RCR, and BAUS guidance, including the requirement for urgent investigation within 24 hours to reduce the likelihood of complications developing and how we can improve patient outcomes with timely and accurate diagnosis by adhering to them. Overall, our recommendations were demonstrated to be impactful; during our evaluation of outcomes in the second cycle, we noted a considerable and statistically significant improvement in the number of patients being investigated with CT KUB per guidelines, as well as in the proportion of scans positive for calculi.

While our results demonstrated compliance with college guidelines regarding the proportion of positive scans for calculi, as well as increased utilisation of CT KUB as the first-line imaging modality, there still remained work to reach the 100% target. We initially proposed implementing a universal system prompt across NUH and primary care providers when requesting urinary tract ultrasounds, as it was the second most used imaging modality in guideline non-compliance. This prompt would encourage clinicians to consider CT KUB as the preferred imaging modality when renal colic is suspected, with links to relevant guidelines for reference. This was in view of a significant and consistent proportion of requests for investigation with ultrasound coming from primary care, accounting for 63.6% (21/33) and 60.0% (12/20) in each respective cycle. However, the use of different operating systems in these settings meant we were not able to action this, and so we could not assess the effectiveness of this recommendation. This presented as one of the limitations faced in our study and could perhaps explain why the goal of 100% compliance remained elusive given how we were not able to adequately address the largest source of ultrasound requests.

Furthermore, the largest source of delays in the investigation of renal colic was also referrals from primary care, standing at 92.0% (23/25) and 87.5% (21/24) for each cycle. We attempted to address this deficit by improving the vetting process, whereby investigations are marked as urgent and a 24-hour time priority allocated accordingly. However, while this reduced the average delay for a scan from 38.04 to 24.65 days, the number of patients who were investigated within 24 hours only improved by one, which was not statistically significant. We recognised a possible confounding factor contributing to this delay being difficulty accessing imaging facilities in the community, due to the lack of streamlined referral and communication pathways where general practitioners may seek advice or immediately refer to the hospital for same-day assessment. 

To reduce delay in future cycles, we aim to establish a pathway that allows direct referrals to the hospital through a dedicated renal colic clinic, to which primary care physicians may refer patients following discussion with a urologist for prompt investigation. On days where this cannot be facilitated, an alternative pathway that enables direct communication between referring general practitioners and radiologists will be established for urgent vetting and scheduling of scans, where findings on the scan will dictate the need for immediate specialist advice and further work-up. Awareness of these changes will be raised through the implementation of local guidelines across practices as an official resource, clarifying the referral process, which will also contain important information regarding radiological investigation standards. This approach may potentially address the difficulty in accessing secondary care facilities and carry the additional benefit of providing accessible reference material to help clinicians stay informed about best practices for investigating suspected renal colic.

Overall, this audit has demonstrated that emphasis placed on clinical history, relayed via investigation requests, can result in a thorough vetting process that allows for a greater proportion of patients to be investigated with CT KUB. This subsequently leads to fewer patients exposed to harmful radiation for the investigation of non-specific abdominal pain with CT imaging when not indicated and a greater likelihood of stone diagnosis, reducing complication rates from missed diagnoses. Although more work needs to be done to achieve targets, with an assessment of future recommendations in a third-cycle audit, this study highlights an important step in addressing the robust investigation of renal colic to improve patient outcomes through prompt diagnosis.

## Conclusions

This quality improvement project at Newham University Hospital has shown that focused interventions, including clinician education and increased awareness of guidelines, can greatly improve adherence to evidence-based standards for managing suspected renal colic. By advocating for the use of CT KUB as the primary investigation tool for renal colic in line with guidance, in conjunction with robust clinical history, we observed a statistically significant improvement in both compliance with standards and the diagnostic accuracy of renal calculi. This improvement is likely to lead to better patient outcomes, as early detection allows for timely treatment and reduces the risk of complications.

While full compliance with all standards was not achieved, it must be remembered that all improvements bring us closer to achieving the highest standards of patient safety and clinical outcomes; our efforts have established a foundation for ongoing progress and further development of actionable recommendations. To maintain momentum towards the 100% target for CT KUB use, we plan to continue regular departmental lectures and display key information on posters for sustained exposure outside of these sessions.

The proposed renal colic clinic pathways will ease access to specialist evaluation and imaging from primary care, with the aim of addressing delays that patients referred from the community faced in both cycles of our study. The effectiveness of this and other recommendations will be evaluated in a third-cycle audit, ensuring ongoing improvement in the prompt investigation of renal colic in the interests of patient safety.
